# *Trib3* Is Developmentally and Nutritionally Regulated in the Brain but Is Dispensable for Spatial Memory, Fear Conditioning and Sensing of Amino Acid-Imbalanced Diet

**DOI:** 10.1371/journal.pone.0094691

**Published:** 2014-04-14

**Authors:** Tiit Örd, Jürgen Innos, Kersti Lilleväli, Triin Tekko, Silva Sütt, Daima Örd, Sulev Kõks, Eero Vasar, Tõnis Örd

**Affiliations:** 1 Institute of Molecular and Cell Biology, University of Tartu, Tartu, Estonia; 2 Department of Physiology, Institute of Biomedicine and Translational Medicine, University of Tartu, Tartu, Estonia; 3 Estonian Biocentre, Tartu, Estonia; 4 Chair of Pathological Physiology, Institute of Biomedicine and Translational Medicine, University of Tartu, Tartu, Estonia; Chiba University Center for Forensic Mental Health, Japan

## Abstract

Tribbles homolog 3 (TRIB3) is a mammalian pseudokinase that is induced in neuronal cell cultures in response to cell death-inducing stresses, including neurotrophic factor deprivation. TRIB3 is an inhibitor of activating transcription factor 4 (ATF4), the central transcriptional regulator in the eukaryotic translation initiation factor 2α (eIF2α) phosphorylation pathway that is involved in the cellular stress response and behavioral processes. In this article, we study the expression of *Trib3* in the mouse brain, characterize the brain morphology of mice with a genetic ablation of *Trib3* and investigate whether *Trib3* deficiency alters eIF2α-dependent cognitive abilities. Our data show that the consumption of a leucine-deficient diet induces *Trib3* expression in the anterior piriform cortex, the brain region responsible for detecting essential amino acid intake imbalance. However, the aversive response to leucine-devoid diet does not differ in *Trib3* knockout and wild type mice. *Trib3* deletion also does not affect long-term spatial memory and reversal learning in the Morris water maze and auditory or contextual fear conditioning. During embryonic development, *Trib3* expression increases in the brain and persists in the early postnatal stadium. Neuroanatomical characterization of mice lacking *Trib3* revealed enlarged lateral ventricles. Thus, although the absence of *Trib3* does not alter the eIF2α pathway-dependent cognitive functions of several areas of the brain, including the hippocampus, amygdala and anterior piriform cortex, *Trib3* may serve a role in other central nervous system processes and molecular pathways.

## Introduction

TRIB3 (also known as TRB3, NIPK and SKIP3) is a kinase-like protein (pseudokinase) that was first isolated as a gene that is strongly induced during neuronal cell death caused by nerve growth factor deprivation or disruption of calcium homeostasis [1], [2]. Further studies revealed that the upregulation of *Trib3* during cellular stress is mediated by the binding of activating transcription factor 4 (ATF4) to the *Trib3* promoter [3], [4]. In mammalian cells, the response to diverse types of cellular stress converges on a single biochemical event – the phosphorylation of eukaryotic translation initiation factor 2α (eIF2α) at serine 51 – that inhibits general translation but selectively increases the translation rate of ATF4, leading to the activation of a multi-faceted stress response gene expression program that is coordinated by ATF4 [5], [6]. Through the four known eIF2α kinases (GCN2, PERK, PKR and HRI), the eIF2α–ATF4 pathway is activated in stress situations such as amino acid or glucose deficiency, unfolded protein accumulation in the endoplasmic reticulum and oxidative damage [5], and, in accordance with the studies of the *Trib3* promoter, these stresses are also characterized by the marked induction of *Trib3* in different types of cells [3], [4], [7]–[10]. The TRIB3 protein is able to act as an inhibitor of ATF4 by directly binding to it [2], [11]. Therefore, the activation of the *Trib3* promoter by ATF4 constitutes a negative feedback mechanism for regulating the activity of the eIF2α–ATF4 pathway [3], [4], [8], [12].

In the brain, the phosphorylation of eIF2α participates in several behavioral processes. Animals are unable to synthesize a subset of amino acids, termed essential amino acids (EAA), and thus need to acquire EAAs from their diet. Omnivores that have a number of different food sources available need to balance their intake of different foods in order to obtain EAAs in the correct proportions. When fed an otherwise complete diet lacking a single EAA, animals including mice and rats will promptly, within the course of one meal, develop an aversive reaction towards the food, which involves substantially limiting the consumption of the food and foraging for alternative dietary sources (reviewed in [13]). This innate reaction does not depend on the gastrointestinal tract, or the senses of taste and smell, but rather on the sensing of blood amino acid levels by the anterior piriform cortex (APC) region of the brain [13]. Following consumption of an EAA-deficient meal, eIF2α is phosphorylated in the APC by GCN2, which is sensitive to intracellular levels of uncharged tRNA molecules, and mice that lack GCN2 fail to reject an EAA-imbalanced diet [14], [15]. However, the mechanisms acting downstream of eIF2α to regulate feeding behavior are currently uncertain.

The eIF2α–ATF4 pathway is also involved in hippocampal long-term memory formation, which is necessary for spatial learning and contextual fear conditioning. Following behavioral training, eIF2α phosphorylation in the hippocampus decreases, and, in mouse models with reduced phospho-eIF2α and ATF4 levels, the threshold for eliciting hippocampus-dependent learning is lowered [16], [17]. Conversely, genetically increasing the amount of eIF2α phosphorylation in the hippocampus to a level that does not inhibit general translation, but does induce ATF4, impairs hippocampal memory [18]. In line with this, the induction of late long-term potentiation, a putative cellular model of learning, is prevented by the pharmacological inhibition of eIF2α dephosphorylation in wild type hippocampal slices but is unaffected in slices from ATF4 knockout mice [17]. Thus, hippocampal long-term memory formation appears to occur *via* the downregulation of the phospho-eIF2α–ATF4 axis in response to behavioral training, but how this process might be influenced by endogenous modulators of ATF4 activity is unknown.

TRIB3 is a target gene and inhibitor of the eIF2α–ATF4 pathway in cell cultures, but its role and expression regulation in the brain are unclear. In the present work we characterize the expression of *Trib3* in the adult mouse brain and during mouse brain development, and, by utilizing a *Trib3* knockout mouse line, study whether TRIB3 has an effect on the behavioral responses that are mediated by the phosphorylation of eIF2α and examine the brain morphology of mice lacking *Trib3*.

## Materials and Methods

### Animals, feeding and diets

The *Trib3* knockout mouse line used in this study was generated by us by introducing a targeted deletion of the entire *Trib3* protein coding region, and is based on the C57BL/6J mouse strain genetic background [19]. Mice were genotyped for knockout and wild type *Trib3* alleles by PCR. The animals were maintained on a 12-hour light/12-hour dark cycle, and experimental procedures were performed during the light phase. Access to food and water was provided *ad libitum*, with the exception of experiments that involved an overnight period of fasting (described below).

In indicated experiments, overnight fasting was used to synchronize feeding between individual mice. During the light phase, food was available *ad libitum*. At the end of the light phase, the remaining food was removed from the feeder and the cage bedding was replaced to avoid the consumption of feed pellet crumbs and to minimize coprophagy. After the dark phase, *ad libitum* access to food was restored. Access to water was not restricted during the fasting period.

To study dietary essential amino acid limitation, synthetic diets composed of purified ingredients were used (manufactured by Research Diets, New Brunswick, NJ). The diets contained free L-amino acids as the sole source of dietary amino acids. A nutritionally complete diet containing an entire complement of amino acids was used for training and as the experimental control diet, and a corresponding diet lacking the essential amino acid leucine was used to evoke leucine deficiency. The composition of the leucine-devoid diet was adjusted with carbohydrate. The full composition of the diets used for leucine deprivation experiments is presented in Table S1. For other experiments, animals were maintained on standard commercial rodent chow.

All animal procedures were performed in accordance with the guidelines of the European Union and were approved by the Estonian National Board of Animal Experiments (resolutions number 83, 25.06.2007; 82, 25.08.2011; 8, 06.05.2013). All efforts were made to minimize suffering of animals during experimental procedures.

### Total RNA isolation, RT-qPCR and RT-PCR

To study adult brain gene expression during leucine deficiency, four-month-old male wild type C57BL/6J mice were trained with synthetic nutritionally complete control diet *ad libitum* for one week, then fasted overnight and randomly provided either the leucine-deficient diet (Leu−) or the control diet (Leu+). Procedures were performed in an alternating order of Leu− and Leu+ group individuals. After 6 h of access to the experimental diet *ad libitum*, the mice were sacrificed by cervical dislocation and their brains were immediately dissected. Brain regions were identified according to the mouse brain atlas of Franklin and Paxinos [20]. The anterior piriform cortex (as defined in [20]), the hippocampus (encompassing both the dorsal and ventral hippocampus), a sample of the cerebral cortex (an approximately 3×3 mm region from the center of the frontal lobe, encompassing all neocortical layers) and the cerebellum (whole) were excised on ice and immediately frozen in liquid nitrogen. To quantify gene expression during mouse development, total RNA was extracted from excised embryonic or neonatal brains (excluding the cerebellum) of the indicated age using TRIzol (Invitrogen). Total RNA was isolated from the APC using the RNeasy Micro kit and from the other adult brain regions using the RNeasy Mini kit (both from Qiagen). Samples were homogenized with a glass-Teflon homogenizer, and on-column DNase digestion was performed according to the manufacturer's recommendations.

Total RNA concentration was determined spectrophotometrically with NanoDrop 1000 (Thermo Scientific), and 0.5 µg of total RNA was used in 10 µl first-strand cDNA synthesis reaction. The developmental brain cDNA series was synthesized with SuperScript III reverse transcriptase (Invitrogen), and the adult brain region cDNA series was synthesized with RNase H-minus M-MLV reverse transcriptase (Solis BioDyne, Estonia). Real-time PCR quantification of *Trib3* mRNA and ribosomal protein L7a (*Rpl7a*) mRNA, which was used as the endogenous reference gene for expression normalization, was performed as described previously [19]. RT-qPCR analysis of *Trib1* and *Trib2* expression was performed as for *Trib3*, using primers with the following sequences: *Trib1* mRNA: 5′-GCTCGGCTCTTCAAGCAGAT-3′ (sense) and 5′-GCTGGGCAGCCATGTTTATC-3′ (anti-sense), *Trib2* mRNA: 5′-TGACCTCAAGCTGCGGAAAT-3′ (sense) and 5′-TAACTGCCGCTGGTGTTCAA-3′ (anti-sense). For end-point RT-PCR, the sequences of the primers used are as follows: *Trib3* mRNA: 5′-GGCCTTATATCCTTTTGGAACGA-3′ (sense) and 5′-CGCTGGCAGGGTACACCTT-3′ (anti-sense), *Gapdh* mRNA: 5′-TGTGTCCGTCGTGGATCTGA-3′ (sense) and 5′-TTGCTGTTGAAGTCGCAGGAG-3′ (anti-sense). The RT-PCR products were analyzed by gel electrophoresis in agarose gel.

### Aversive response to amino acid-deficient food

The protocol used for studying the effect of amino acid-devoid diet on food intake was adapted from a previously published article [15]. Four-month-old mice were used in the experiment, and for both *Trib3* ^+/+^ and *Trib3* ^−/−^ genotypes, the experimental group contained four females and three males. The animals were arranged in a random order and the experimenter was blind to mouse genotype. Mice were trained for one week to the novelty of the synthetic control diet, single housing and food deprivation during the dark phase (overnight fasting). Single housing was used to enable the determination of food consumption by weighing the food pellets remaining in the feeder (visible food pellet crumbs found inside the cage were also included in the measurement). In the days following the training period, the animals' food consumption was measured at set time-points throughout the light phase. On the first three days, the intake of the control diet was monitored, and on the next three days, leucine-deficient diet consumption was investigated. For every animal, the consumption of the leucine-devoid diet was compared to the amount of complete diet consumed by the same animal, which serves to control for individual variations in food intake that are independent of the dietary conditions investigated.

### Morris water maze

The experimental groups consisted of three-month-old littermate *Trib3* ^+/+^ and *Trib3* ^−/−^ mice, and both groups were sex-balanced (*Trib3* ^−/−^ group: 8 males and 8 females; *Trib3* ^+/+^ group: 5 males and 5 females). Animal housing was not divided by genotype. To perform the experimental procedures, the animals in each cage were assigned a random order that was kept consistent over the course of the experiment. The experimenter was blind to mouse genotype. The pool diameter was 150 cm, and the water was kept at room temperature and rendered opaque by the addition of a small amount of non-toxic white putty. The platform diameter was 16 cm and its top surface was approximately 1 cm below the surface of the water. One day before the start of training, the mice were placed into the pool (with no platform) for 60 seconds to habituate them with swimming and handling. Mice were trained four times per day at intervals of approximately 45 minutes for four consecutive days. In each training trial, the animal was allowed to swim until it found the platform, but not for more than 60 seconds. If the platform was not found after 60 seconds, the mouse was guided to the platform by the experimenter. After arriving on the platform, the mouse was left there for 15 seconds before being picked up. Four different start positions, located beside the side wall of the pool at 90° intervals, were used once per day by every mouse. For each training day, the start position order was permutated for each mouse. One day after the end of the training phase, a probe trial was performed by removing the platform from the pool and allowing the mice to search for 60 seconds. An automated video tracking system was used to monitor the swimming trajectory and time (TSE Systems GmbH, Germany). During training trials, the time required for finding the platform (escape latency) was recorded, and for probe trials, the time spent searching in each pool quadrant was recorded. Following the probe trial, reversal training was started on the same day and performed for two consecutive days. For reversal training, the platform was positioned at the quadrant opposite of the platforms' original location and the training protocol corresponded to that of the initial training. The reversal probe trial was performed one day after the end of the reversal training phase.

### Fear conditioning

The fear conditioning experiments were performed using the same groups of *Trib3* ^+/+^ and *Trib3* ^−/−^ mice that were previously used in the Morris water maze, after a resting period of approximately one month. The housing arrangement and temporal order of animals in the experimental procedures was the same as in the Morris water maze. The experimenter was blind to mouse genotype. To induce leucine deficiency during the experiment, mice were first habituated to the synthetic control diet *ad libitum* for 12 days, and then deprived of food overnight on the night before the training day. Starting from the morning of the experimental training day, mice were fed the synthetic diet lacking leucine *ad libitum*. The fear conditioning training was performed five hours after the initiation of leucine deprivation. For training, a mouse was placed into the training chamber (TSE Systems GmbH), allowed to acclimatize to the context for 120 seconds, followed by two pairings of a tone (30 seconds, 75 dB, 10 kHz) with a co-terminating foot shock (2 seconds, 0.5 mA, constant current). The two pairings were separated by a 120-second pause, and mice remained in the training chamber for 15 seconds after the last pairing. Twenty-four hours after training, contextual fear conditioning was assayed by placing the animal into the training chamber for 5 minutes and recording its movement. Two hours later, auditory fear conditioning was assayed by placing the animal into a visually different chamber and recording its movement during a 150-second habituation (pre-tone) phase, followed by a 150-second phase of training tone presentation. The incidence of freezing (immobility except for respiration) during each testing phase was tracked with an automated system (TSE Systems GmbH). After testing, the animals were returned to a standard diet.

### RNA *in situ* hybridization

Preparation of mouse brain sections and their analysis by *in situ* hybridization using digoxigenin-labeled riboprobes was performed as described previously [21]. To generate the template for the probe targeting synaptophysin (*Syp*) mRNA, a 463-bp cDNA fragment was PCR-amplified from mouse brain cDNA using the primers 5′-CCCAAGCTTGGGGGTCAGTTCCGGGTGGT-3′ and 5′-CCGCTCGAGCTTCACATCGGACAGGCCTT-3′ (sense and anti-sense, with *Hin*dIII and *Xho*I restriction sites underlined, respectively) and cloned into the pBlueScript KS+ vector (Stratagene).

### Lateral ventricle size measurement

Lateral ventricle size was studied from PFA-fixed coronal sections by measuring the area of the lateral ventricles from a microphotograph of the section, using Adobe Photoshop software, with the experimenter blind to genotype. The areas of the left and right lateral ventricle were summed for each individual. For adult mice, the section selected for the measurement was located at the level 0.1–0.22 mm posterior to bregma (figures 32 and 33 in [20]), and the anterior–posterior location of the section was determined based on the dorsal part of the third ventricle, the anterior part of the anterior commissure and the anterior part of the paraventricular thalamic nucleus. The adult study groups consisted of age- and sex-matched *Trib3* ^+/+^ and *Trib3* ^−/−^ individuals (4 males and 3 females for each genotype) with an age of 5.5–7 months. For 9-day-old mice, the section selected for the measurement corresponded to 0.02–0.1 mm posterior to bregma (figures 31 and 32 in [20]), and the anterior–posterior location of the section was determined based on the anterior part of the anterior commissure and the shape of the dorsal part of the third ventricle. Both the adult and juvenile animals were maintained in standard laboratory animal husbandry conditions and were not subjected to prior experimental procedures.

### Southern blot

Ten micrograms of mouse genomic DNA was digested with *Nco*I (Fermentas, Lithuania) and separated by gel electrophoresis in 1% agarose gel. DNA fragments were transferred onto Hybond-N+ positively charged nylon membrane (Amersham) according to the manufacturer's recommendations. To prepare the probe, a 1.2-kb DNA fragment corresponding to the genomic region immediately downstream of the *Trib3* termination codon was purified and radiolabeled in a random-primed labeling reaction containing 50 µCi [α-^32^P]-dCTP (Hartmann Analytic GmbH, Germany) using the DecaLabel DNA labeling kit (Fermentas). Autoradiography of the hybridized probe was performed by storage phosphor imaging on a Typhoon Trio imager (GE Healthcare).

### Statistical analysis

Data are expressed as the mean ± SEM, and group sizes are stated in the figure legends. To analyze the effect of leucine deficiency on gene expression in different brain regions, gene expression in leucine-devoid diet-fed and control diet-fed individuals was compared for each studied brain region with the two-tailed *t* test, and the resulting *P*-values were corrected for multiple comparisons with the Holm–Bonferroni method, using R software (version 3.0.2; The R Foundation for Statistical Computing). Repeated measures ANOVA was used to compare amino acid-deficient diet rejection between genotypes and Morris water maze escape latency between genotypes, using the Statistica software package (version 8.0; StatSoft). Gene expression changes between different stages of brain development were analyzed in R by performing one-way ANOVA followed by pairwise comparisons of all group means with correction for multiple testing using the Holm–Bonferroni method. Lateral ventricle size was compared between genotypes with the Mann–Whitney *U* test, using Statistica. For experiments not specified above, comparisons between groups were performed with the two-tailed *t* test. For all analyses, *P*<0.05 was considered statistically significant.

## Results

### Consumption of essential amino acid-deficient diet induces *Trib3* mRNA expression in the anterior piriform cortex

**Figure 1:**
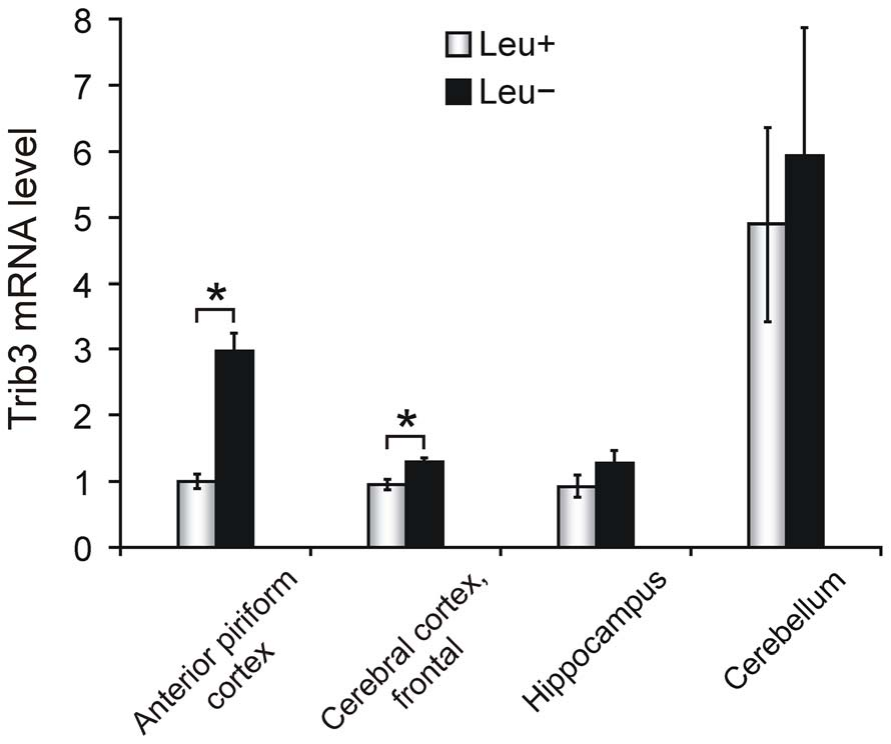
*Trib3* is upregulated in the mouse anterior piriform cortex in response to leucine-deficient diet. Adult wild type mice consumed either a diet lacking leucine (Leu−; n = 5) or a corresponding control diet containing leucine (Leu+; n = 5), and, after 6 h of feeding, *Trib3* expression in the indicated brain regions was quantified by RT-qPCR. The results are presented as the mean ± SEM, and expressed relative to the level of *Trib3* mRNA in the anterior piriform cortex of the control diet (Leu+) group. *P<0.05 comparing leucine-starved and control diet-fed groups.

When mice are fed a diet lacking an EAA, the concentration of that EAA is reduced in the blood and GCN2-dependent phosphorylation of eIF2α ensues in the APC, the region containing the brains' chemosensor for EAA deprivation [14], [15]. To determine whether *Trib3* is upregulated in the brain in response to EAA deficiency, adult mice were habituated to a nutritionally complete synthetic diet *ad libitum* for one week, which was followed by overnight fasting to synchronize feeding before being provided either a synthetic diet lacking the EAA leucine (Leu−) or the corresponding leucine-containing complete diet (control; Leu+). After 6 h, the mice were sacrificed, and RT-qPCR was used to quantify *Trib3* expression in various brain regions. In the complete diet-fed mice, the *Trib3* expression level is very similar in the frontal cerebral cortex, the APC and the hippocampus, while in the cerebellum it is approximately 5-fold higher (Figure 1). In leucine-deprived mice, *Trib3* mRNA abundance is increased 3-fold in the APC, compared to Leu+ mice, and it is also slightly but statistically significantly elevated in the cerebral cortex, while no significant change in *Trib3* expression is detectable in the hippocampus or cerebellum (Figure 1).

In addition to *Trib3*, there are two other *Drosophila Tribbles* homologs in mammals, *Trib1* and *Trib2*. RT-qPCR analysis of *Trib1* and *Trib2* expression in the adult mouse brain was performed in the same brain regions and dietary conditions as for *Trib3*. The results show that in complete diet-fed mice, *Trib1* expression level does not vary between the APC, the cerebral cortex and the cerebellum, and is approximately 50% lower in the hippocampus compared to the other studied regions (Figure S1A), while the level of *Trib2* expression is uniform in all of the four studied brain regions (Figure S1B). Neither *Trib1* nor *Trib2* demonstrate a significant change in expression level in any of the studied brain regions in response to the consumption of leucine-deficient diet (Figure S1A and B). Thus, in comparison with the other *Tribbles* homologs, *Trib3* displays a unique expression pattern in the adult mouse brain, with elevated basal expression in the cerebellum compared with the cerebrum, and *Trib3* is the only gene in the *Tribbles* homolog family that is induced in the APC by amino acid deficiency.

### Deletion of *Trib3* does not influence the rejection of amino acid-imbalanced diet

**Figure 2:**
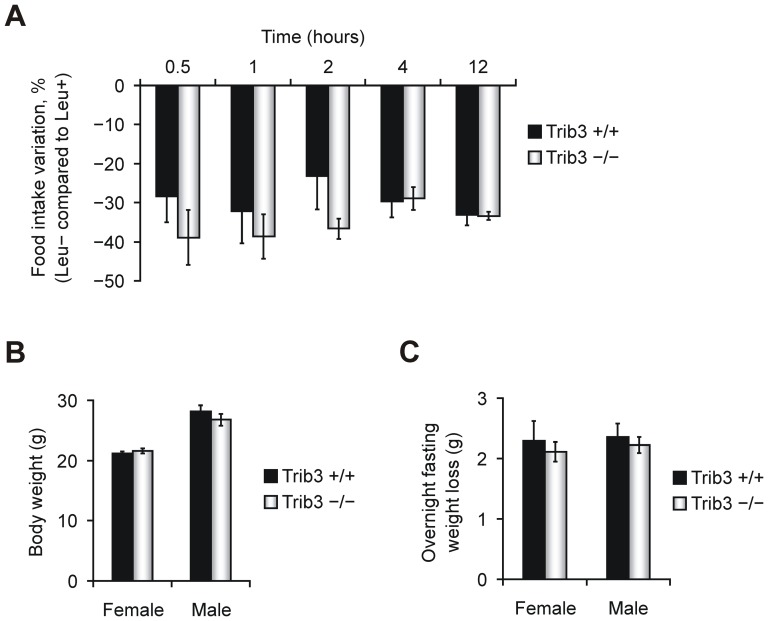
Rejection of amino acid-imbalanced diet in mice is not influenced by the deletion of *Trib3*. (**A**) Consumption of a diet lacking the essential amino acid leucine (Leu−), compared to the consumption of a corresponding nutritionally complete control diet (Leu+). Adult *Trib3* ^+/+^ and *Trib3* ^−/−^ (n = 7 per genotype) mice were trained with food deprivation during the dark phase, and, during the light phase, food intake was measured for each animal at the indicated time-points by weighing the remaining food. For each animal, the intake of Leu− diet was compared to that of the Leu+ diet, and the average difference in the consumption of the Leu− diet relative to the Leu+ diet is expressed in percent ± SEM for each genotype. (**B**) Body weight and (**C**) body weight loss due to overnight food deprivation do not differ between *Trib3*-deficient and wild type adult mice. For B and C, four-month-old group-housed *Trib3* ^+/+^ (n = 5 for both males and females) and *Trib3* ^−/−^ (n = 8 for both males and females) mice were maintained on the Leu+ diet *ad libitum* for two weeks to determine their diet-habituated body weight, followed by a single iteration of overnight fasting. The data in B and C are presented as the group means ± SEM.

Because the aversive response to an amino acid-deficient diet is dependent on the modulation of eIF2α phosphorylation [14], [15], and because *Trib3* was revealed to be induced by EAA depletion in the EAA-sensitive APC region of the brain (Figure 1) and is known to regulate eIF2α–ATF4 pathway activity during amino acid limitation in cell cultures [12], we sought to explore the importance of *Trib3* for the sensing of amino acid-imbalanced diet in mice. We have recently generated a *Trib3*-deficient mouse line by introducing a targeted deletion of the protein coding region of the *Trib3* gene, as detailed in Figure S2A and B. As expected, RT-PCR analysis of *Trib3* expression in mouse brain readily detects *Trib3* mRNA in *Trib3* ^+/+^ and *Trib3* ^+/−^ individuals, while *Trib3* mRNA is undetectable in *Trib3* ^−/−^ littermates (Figure S2C). To study the aversion to amino acid-deficient diet, *Trib3* ^+/+^ and *Trib3* ^−/−^ mice were fasted 12 h overnight and presented with either Leu+ or Leu− diets during the day, and food intake was measured at 0.5 h to 12 h time-points. As depicted in Figure 2A, both *Trib3* ^+/+^ and *Trib3* ^−/−^ mice demonstrate a similar and substantial rejection of Leu− food starting from 0.5 h, and, at the later time-points (4 h and 12 h), where the intra-group variation is lower, the consumption of Leu− diet is decreased by approximately 30% compared to Leu+ for both genotypes. The proportion of food intake repression for EAA-deficient diet is comparable to results that other researchers have obtained for wild type mice [15]. In both males and females, the body weight of littermate wild type and *Trib3*-deficient mice habituated to the synthetic complete diet (Leu+) is similar (Figure 2B), as is the amount of weight lost due to overnight fasting (Figure 2C). Thus, the loss of *Trib3* does not alter the self-restriction behavior of mice in response to amino acid-insufficient food.

### *Trib3* is dispensable for spatial and reversal learning in the Morris water maze

**Figure 3:**
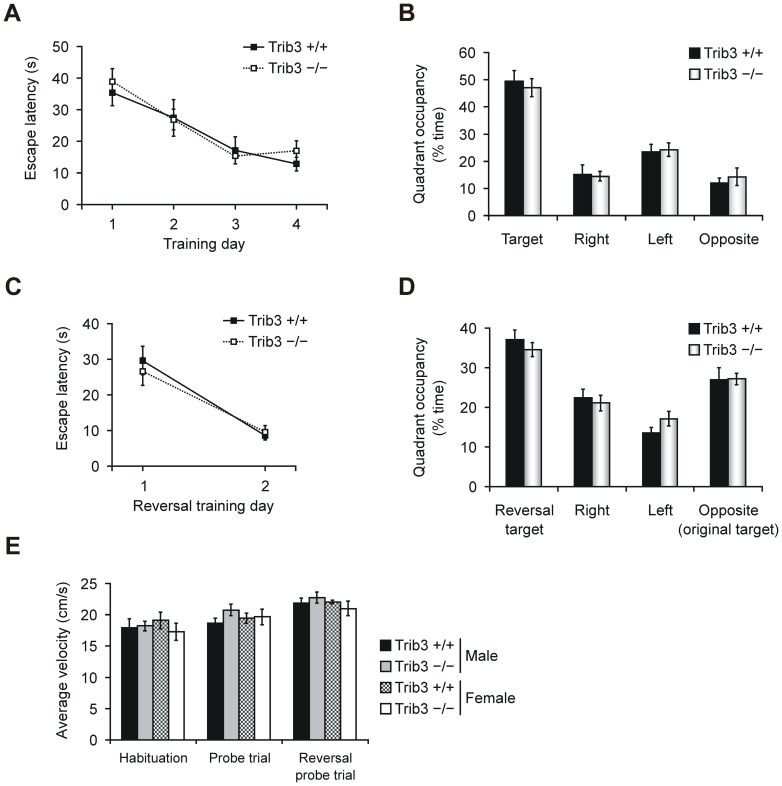
Long-term spatial memory and reversal learning ability in the Morris water maze is not altered in *Trib3*-deficient mice. Data are means ± SEM from littermate *Trib3* ^−/−^ (n = 16) and *Trib3* ^+/+^ (n = 10) mice. (A) Escape latencies during four days of hidden-platform training performed at four trials per day. (B) Pool quadrant occupancy in a probe trial performed 24 h after the completion of training. The submerged platform was removed from the pool and the swim trajectory of mice was monitored for 1 min. The pool quadrant that previously contained the platform is designated the target quadrant, and the time spent in each quadrant of the pool is presented as percent of the total search time. (C) Reversal training escape latencies during two days of training performed at four trials per day. For reversal training, the hidden platform was repositioned to the pool quadrant opposite of the initial platform location. (D) Results of a probe trial performed 24 h after reversal training. The probe trial was carried out as in B. The reversal target denotes the pool quadrant that contained the platform during the reversal trainings trials. (E) Swimming speed at different stages of the experiment. The mean speed ± SEM from 60-second swimming sessions with no platform are shown. The habituation session was performed one day before the start of training. For *Trib3* ^+/+^, n = 5 per sex, and for *Trib3* ^−/−^, n = 8 per sex.

Excessive hippocampal ATF4 activity resulting from eIF2α phosphorylation has been associated with impaired spatial memory and learning [17], [18], and the hippocampus also expresses *Trib3* (Figure 1). To examine the role of *Trib3* in long-term spatial memory, we studied the performance of *Trib3* ^+/+^ and *Trib3* ^−/−^ mice in the Morris water maze, a hippocampus-dependant task in which mice learn to escape from opaque water onto a submerged platform by following spatial cues [22]. As shown in Figure 3A, *Trib3*-deficent mice and their wild type counterparts demonstrate similar and consistent improvement in the time required to find the hidden platform (escape latency) over the first three training days, and no further improvement is displayed by either genotype on training day four. One day after the last training day, a probe test was performed by removing the platform and allowing the mice to search for one minute. Both genotypes exhibited a strong preference for the quadrant of the pool that previously contained the platform (target quadrant), spending nearly 50% of the time there, indicating that no significant differences exist between the genotypes in spatial learning ability (Figure 3B).

The Morris water maze can also be used to study spatial reversal learning by repositioning the platform and challenging the mice to learn the new platform location. Recently, it has been published that mice with a forebrain-specific postdevelopmental disruption of the eIF2α kinase PERK have unaltered learning in the Morris water maze but are impaired in reversal learning, implicating the eIF2α phosphorylation pathway in behavioral flexibility [23]. Therefore, we also studied reversal learning ability in *Trib3* knockout mice. For this experiment, the hidden platform was relocated to the opposite quadrant. As indicated in Figure 3C, *Trib3* ^+/+^ and *Trib3* ^−/−^ mice display similar escape latency of nearly 30 seconds on the first day of reversal training, and, for both genotypes, the escape latency improves drastically on the second reversal training day, with *Trib3*-deficient as well as wild type individuals swimming to the hidden platform in approximately 10 seconds on average. In accordance with this result, no significant differences are observable between *Trib3* ^+/+^ and *Trib3* ^−/−^ mice in the quadrant occupancy pattern of a probe trial performed after reversal training (Figure 3D). The swimming speeds exhibited in 60-second sessions at different phases of the experiment are similar for both genotypes (Figure 3E), indicating that locomotor ability in the Morris water maze is not compromised by the lack of *Trib3*.

### *Trib3*-deficient mice display normal contextual and auditory fear conditioning

**Figure 4:**
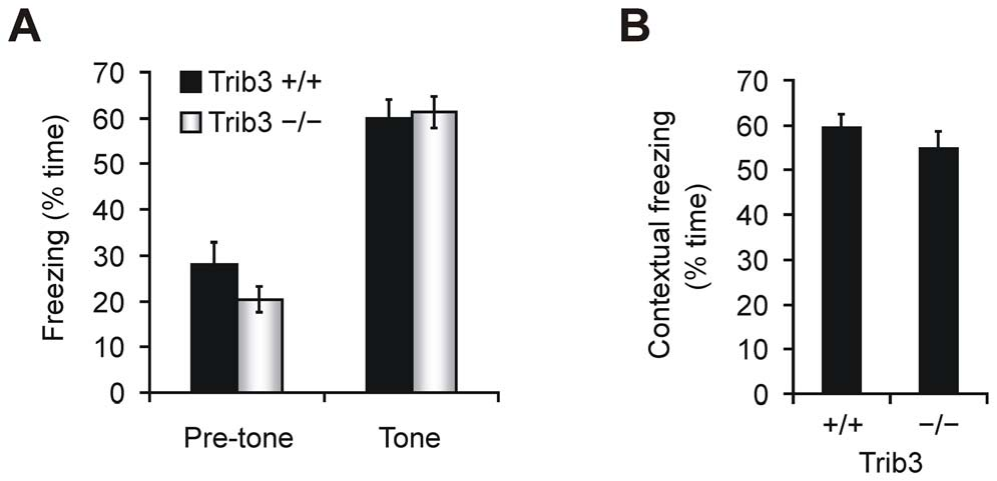
Contextual and auditory fear conditioning is unaffected in mice with a targeted disruption of *Trib3*. Data are means ± SEM from littermate *Trib3* ^−/−^ (n = 16) and *Trib3* ^+/+^ (n = 10) mice. The testing of fear memory was performed approximately 24 h after training, and the animals were fed a leucine-deficient diet starting from the morning of the training day. (**A**) Auditory fear conditioning in mutant and wild type mice. For testing, the mice were placed into a chamber differing from the training chamber, and their movement was monitored during a habituation phase (pre-tone) and during the presentation of the auditory cue (tone). The percentage of time spent freezing is presented for each phase. (**B**) Contextually triggered freezing in *Trib3* ^+/+^ and *Trib3* ^−/−^ mice. To assess contextual fear conditioning, the activity of the mice was monitored upon being returned to the training chamber, and the fraction of time spent freezing is expressed in percent.

In a fear conditioning experiment, animals learn to associate an aversive stimulus with a neutral stimulus or context [24], and long-term fear memory is known to involve eIF2α but the genes acting downstream of eIF2α are uncertain [16], [17], [25]. We trained *Trib3* knockout and wild type mice with two pairings of tone with co-terminating foot shock, and to induce *Trib3*, the animals were fed a leucine-deficient diet *ad libitum* from the morning of the training day until the end of the measurements (performed one day after training). Auditory fear conditioning, which requires the amygdala but not the hippocampus [24], was tested by presenting the training tone in a chamber different from the training chamber and measuring the amount of time spent freezing, an indicator of fear. *Trib3* ^+/+^ and *Trib3* ^−/−^ mice demonstrated a similar robust increase in freezing time during the tone period compared to the pre-tone period (Figure 4A). Contextual fear conditioning, which requires both the amygdala and the hippocampus [24], was tested by placing the animal into the training chamber and tracking its movement over the course of 5 minutes. The amount of time spent freezing did not differ significantly between mice lacking *Trib3* and their wild type counterparts, with both genotypes freezing for more than 50% of the time spent in the chamber (Figure 4B). Thus *Trib3* does not affect long-term fear memory.

### *Trib3* expression increases during embryonic mouse brain development

**Figure 5:**
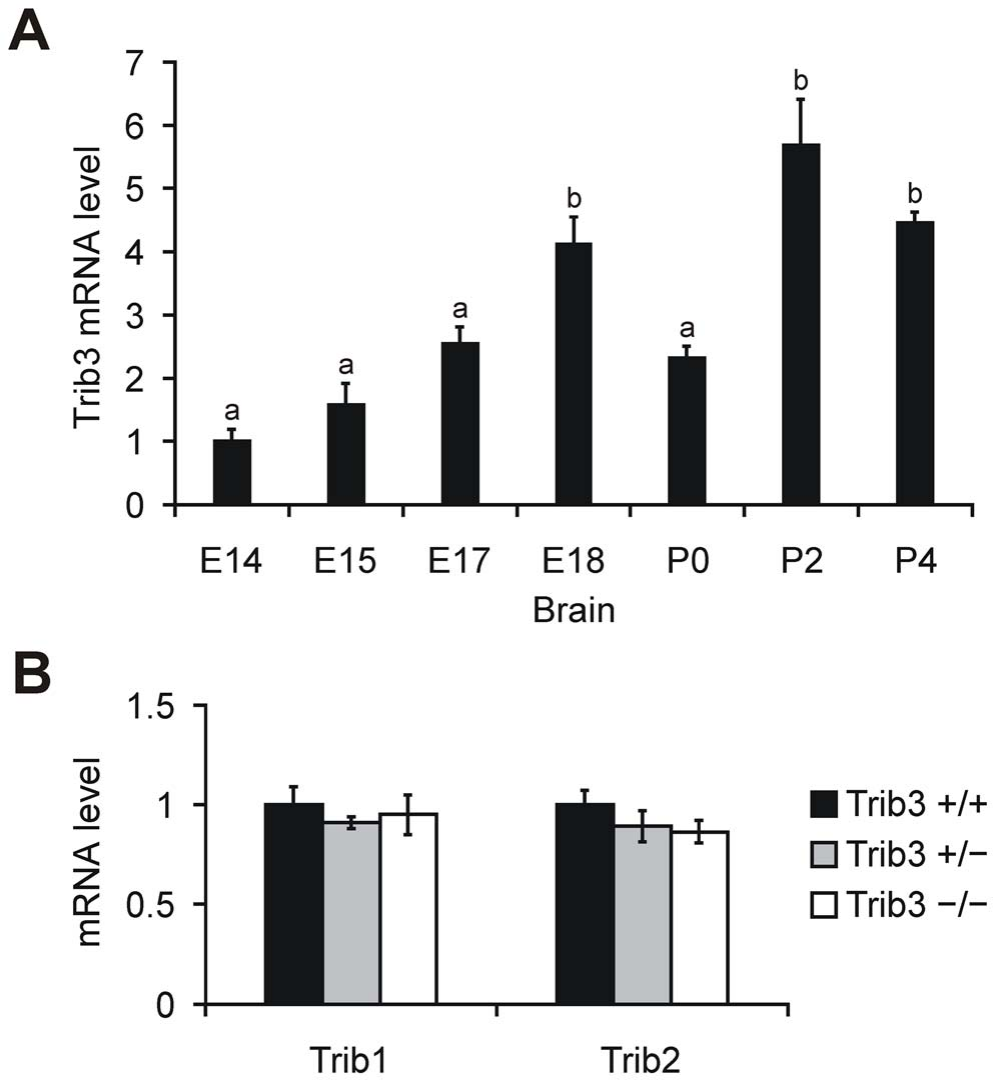
*Trib3* is developmentally regulated in the mouse brain. (**A**) RT-qPCR quantification of *Trib3* expression in wild type C57BL/6J mouse brains at embryonic day (E) 14, 15, 17 and 18, and at postnatal day (P) 0, 2 and 4. The mean *Trib3* expression level ± SEM at the indicated age is presented relative to the level of *Trib3* expression at E14 (n = 7 for E17, E18 and P0, n = 6 for E15, n = 5 for E14 and P2, and n = 3 for P4). Means marked with the same letter are not significantly different at the 5% significance level. (**B**) Lack of *Trib3* does not lead to altered expression of other *Tribbles* family genes in the P3 mouse brain. RT-qPCR was used to determine the level of *Trib1* and *Trib2* mRNA expression in littermate *Trib3* ^+/+^, *Trib3* ^+/−^ and *Trib3* ^−/−^ mice (n = 5 per genotype). For both genes, the mean ± SEM is presented relative to the level of expression in *Trib3* ^+/+^ mice.

In *Drosophila*, the Tribbles protein is expressed in the embryo and participates in early embryonic development [26]–[28]. Therefore, we sought to examine the expression of *Tribbles* homologs in the developing mammalian brain. RNA was extracted from mouse brains ranging from embryonic day (E) 14 to postnatal day (P) 4, and the abundance of *Trib1*, *Trib2* and *Trib3* mRNA was determined by RT-qPCR. The results show that *Trib3* expression increases steadily from E14 to E18, by approximately 4-fold in total (Figure 5A). After birth (P0), the level of *Trib3* mRNA is decreased by approximately 50% compared to E18, however, this decrease appears to be transient, as a resumption of elevated *Trib3* expression is evident at over following days, with a peak at P2 reaching nearly 6-fold higher than E14 (Figure 5A). Unlike *Trib3*, the changes in *Trib1* and *Trib2* expression over the course of mouse brain development are very mild, with both genes demonstrating expression variations of less than 2-fold from E14 to P4 (Figure S3A and B). To study whether the loss of *Trib3* expression affects the mRNA levels of the other *Tribbles* homologs in the developing brain, we quantified the expression levels of *Trib1* and *Trib2* in littermate *Trib3* ^+/+^, *Trib3* ^+/−^ and *Trib3* ^−/−^ mouse brains at P3. The results, presented in Figure 5B, show a lack of compensatory regulation of *Trib1* or *Trib2* expression in the developing brain in response to *Trib3* deletion. These results indicate a possible role for *Trib3* in the pathways regulating brain development.

### Enlarged lateral ventricles in *Trib3* ^−/−^ mice

**Figure 6:**
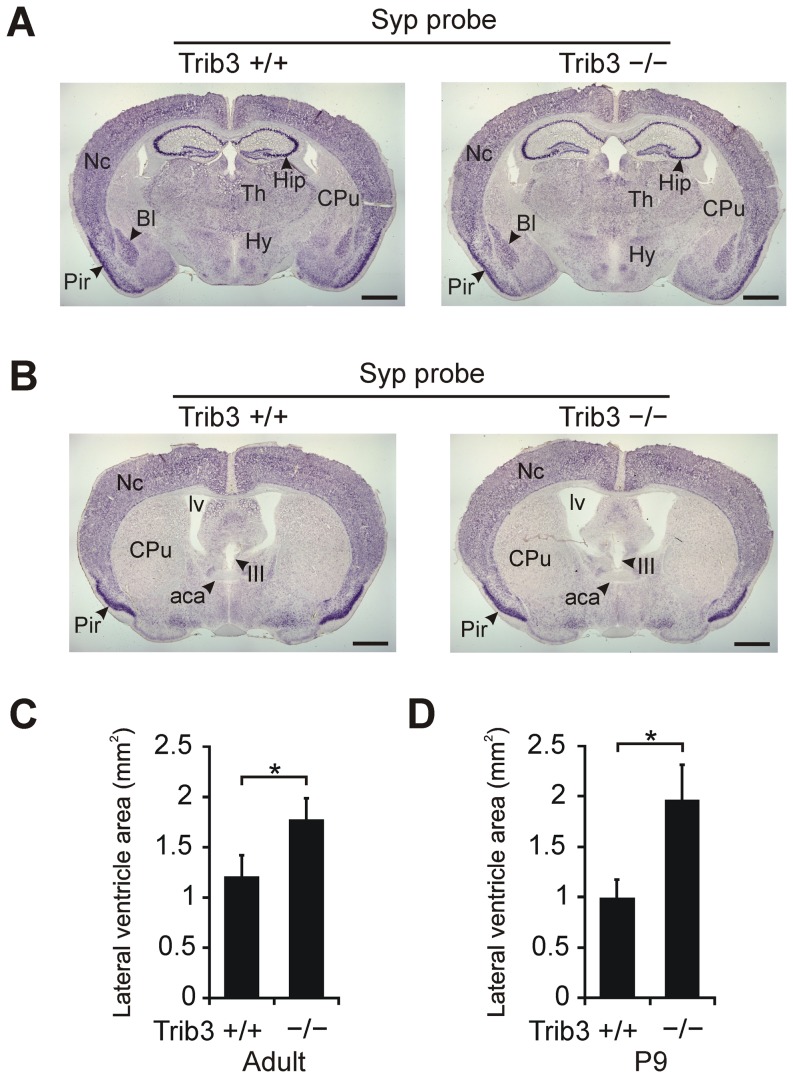
Gross brain morphology of *Trib3* knockout (*Trib3* ^−/−^) and corresponding wild type (*Trib3* ^+/+^) mice. (**A** and **B**) Representative adult mouse brain coronal sections are shown hybridized with a digoxigenin-labeled RNA probe complementary to mRNA encoding the synaptic vesicle protein Syp. (**C**) Size of lateral ventricles in adult *Trib3* ^+/+^ and *Trib3* ^−/−^ mice (n = 7 per genotype). The area of the lateral ventricles was measured from coronal sections at the level depicted in panel B. (**D**) Size of lateral ventricles at postnatal day 9 (P9) in *Trib3* knockout mice and their wild type littermates (n = 5 per genotype). In C and D, the areas of the left and right lateral ventricle on the coronal section were summed for each mouse, and the mean ± SEM for each genotype is presented. Abbreviations: aca, anterior commissure, anterior part; Bl, basolateral amygdala; CPu, caudate–putamen; Hip, hippocampus; Hy, hypothalamus; III, third ventricle; lv, lateral ventricle; Nc, neocortex; Pir, piriform cortex; Th, thalamus. Scale bar 1 mm. *P<0.05 comparing genotypes.

To study the effect of germline *Trib3* gene inactivation on brain morphology, sections from adult littermate *Trib3* ^+/+^ and *Trib3* ^−/−^ mouse brains were visualized by RNA *in situ* hybridization of *Syp* mRNA, which encodes the synaptic marker synaptophysin [29]. The results show that the expression pattern of *Syp* is similar in *Trib3* ^+/+^ and *Trib3* ^−/−^ individuals, and that the gross morphology of many prominent brain structures is unaltered by *Trib3* deficiency (Figure 6A). However, the lateral ventricles are noticeably enlarged in mice lacking *Trib3* compared to the corresponding wild type mice (Figure 6B). To study this further, lateral ventricle area was measured from coronal brain sections of a group of *Trib3* ^+/+^ and *Trib3* ^−/−^ mice at 5.5–7 months of age. As depicted in Figure 6C, the size of the lateral ventricles is significantly increased, by 47% on average, in adult *Trib3*-deficient mice compared to wild type individuals. Similarly, in juvenile (P9) mice, lateral ventricle size is approximately 2-fold greater in *Trib3* ^−/−^ mice than in corresponding *Trib3* ^+/+^ individuals (Figure 6D). Thus, *Trib3* may serve a function associated with the ventricular system in the brain.

## Discussion

TRIB3 is known to be upregulated in neuronal cell cultures subjected to cell death-inducing stresses [1], [2], [30], but its role and regulation in the brain under physiological conditions have not been investigated previously. In this article, we studied the expression of *Trib3* in the mouse brain during adulthood and development, the brain morphology of *Trib3*-deficient mice, and characterized the behavioral phenotype of *Trib3* knockout mice, including their long-term spatial memory, fear memory and response to amino acid-insufficient food.

In the adult mouse brain, our experiments revealed that *Trib3* is markedly induced in the APC region by dietary EAA insufficiency within 6 hours. Previous knowledge of transcriptional changes in the APC in response to amino acid deficiency is relatively scarce. Consumption of an EAA-incomplete meal leads to the depletion of the limiting amino acid in blood plasma, and, in the brain, the concentration of the limiting amino acid is decreased in the APC, leading to eIF2α phosphorylation [15], [31]. The phosphorylation of eIF2α is coupled to the upregulation of ATF4 [5], which acts as a master transcriptional activator of C/EBP-ATF composite sites [6], a type of stress-sensitive regulatory element. In various continuous cell lines, examination of the *Trib3* promoter has revealed that the upregulation of *Trib3* in response to chemical inducers of endoplasmic reticulum stress and oxidative stress is mediated by the binding of ATF4 to a C/EBP-ATF site [3], [4]. Therefore, it is likely that the mechanism of *Trib3* regulation by nutrients *in vivo* in the brain also proceeds *via* the C/EBP-ATF composite site in the *Trib3* promoter.

Mice carrying a targeted deletion of *Trib3*, generated recently by us [19], have no apparent physical defects, allowing for behavioral testing to be performed. Our experiments uncovered that a lack of *Trib3* does not affect aversion to EAA-imbalanced diet, which is dependent on the APC, long-term spatial memory, which is dependent on the hippocampus, or fear conditioning, which is dependent on the amygdala. These behavioral paradigms require the modulation of eIF2α phosphorylation in the brain, and for long-term memory consolidation, the control of ATF4 levels appears to be the crucial function of phospho-eIF2α [14], [15], [17], [18]. In light of cell culture-based data which demonstrates that TRIB3 provides negative feedback inhibition of ATF4 activity [3], [4], [8], [12], the behavioral test results obtained for *Trib3*-deficient mice are unexpected. It is possible that TRIB3 does not significantly inhibit ATF4 activity in the brain during normal physiology, or that the inhibitory effect of TRIB3 does not extend to the particular aspect of ATF4 function that is necessary for memory formation, which is proposed to be the antagonism of CREB [16]. Alternatively, possible ATF4-dependent or -independent effects of *Trib3* in the brain are masked in *Trib3* ^−/−^ mice by slight alterations in nervous system development or the activation of intracellular signaling mechanisms that are able to compensate for the absence of *Trib3*. The importance of *Trib3* expression in the brain may also be revealed in behavioral responses that are currently unexplored in *Trib3*-deficient mice. In mammals, two additional *Tribbles* homologs, *Trib1* and *Trib2*, are present along with *Trib3*. However, the molecular and physiological functions of the three *Tribbles* homologs have diverged [32], and the ability to interact with ATF4 has only been shown in the case of *Trib3*. This reduces the likelihood that *Trib1* and *Trib2* could compensate for the deletion of *Trib3* in the context of ATF4 activity regulation, and we did not detect altered expression of *Trib1* or *Trib2* in the brain of neonatal *Trib3* knockout mice. Further, our data demonstrate that the expression pattern of *Trib3* in the brain is distinct from that of the other members of the *Tribbles* homolog family. Notably, only *Trib3* was induced in the APC by EAA deprivation and only *Trib3* exhibited prominent upregulation during embryonic brain development.

Our analysis of *Trib3* expression during mouse brain development revealed that the abundance of *Trib3* mRNA in the brain increases from E14 to E18 and remains high in the neonatal brain. During vertebrate brain development, a substantial proportion of newly generated neurons undergo programmed cell death, which is caused in part due to the limited availability of neurotrophic factors [33]. In the mouse forebrain, programmed cell death is prevalent during the period from E12 to E18, encompassing both proliferative and postmitotic neurons [34]. Therefore, it is possible that the induction of *Trib3* during embryonic brain development is related to neurotrophic factor deficiency or neuronal cell death. Consistent with this assumption, *Trib3* is upregulated in neuronally differentiated PC12 cells and superior cervical ganglion neurons in response to nerve growth factor deprivation [1], [35], [36]. In addition to neuronal cells, BV-2 microglial cells also upregulate *Trib3* expression under certain conditions [37], [38]. Thus, the origin of *Trib3* expression during central nervous system development warrants evaluation in future studies.

Examination of the brain morphology of adult and juvenile *Trib3*-deficient mice revealed increased lateral ventricle size compared to mice with wild type *Trib3*. In humans, lateral ventricle size increases with aging and enlarged ventricles are associated with age-related brain disorders, including Alzheimer's disease [39], however, lateral ventricle volume also displays a relatively large amount of variability in healthy, non-elderly humans [40]. Further experiments are needed to study the dynamics of *Trib3* deficiency-related ventricular expansion during mouse brain aging, as well as the neurological, neuroanatomical and behavioural importance of this effect, and to elucidate the mechanism by which the lack of *Trib3* results in enlarged lateral ventricles.

In conclusion, we establish that *Trib3* expression increases in the mouse brain during the progression of embryonic brain development, and, in the adult brain, *Trib3* is induced by dietary essential amino acid deprivation. Nevertheless, mice homozygous for a germline deletion of *Trib3* are normal regarding several aspects of cognitive functioning, including spatial learning and re-learning, fear memory, and the self-restriction of amino acid-deficient diet intake.

## Supporting Information

Figure S1 ***Trib1*** **(A) and** ***Trib2*** **(B) expression levels in various regions of the adult mouse brain.** Wild type mice consumed either a diet lacking leucine (Leu−; n = 5) or a corresponding control diet containing leucine (Leu+; n = 5), and, after 6 h of feeding, gene expression in the indicated brain regions was quantified by RT-qPCR. The results are presented as the mean ± SEM, and expressed relative to the level in the anterior piriform cortex of the control diet (Leu+) group.
(TIF)

Figure S2 **Targeted disruption of the mouse** ***Trib3*** **gene.** (**A**) Schematic representation of the gene targeting strategy used to generate the *Trib3*-deficient allele. Filled and unfilled boxes indicate exonal regions containing the *Trib3* protein coding sequence and mRNA untranslated regions, respectively. The 5′ and 3′ homology arms (3.1 and 1.9 kb, respectively) for the homologous recombination event were selected to flank the genomic region corresponding to the *Trib3* protein coding sequence. The homology regions were PCR-amplified and cloned into a targeting vector that contained a phosphoglycerate kinase promoter-driven neomycin resistance cassette (pgk-NeoR) for positive selection and a thymidine kinase promoter-driven diphtheria toxin A expression cassette (tk-DTA) for negative selection. The *Nco*I restriction sites that generate the genomic DNA fragments detected in panel B are indicated by unfilled vertical arrowheads. (**B**) Verification of the targeted disruption by Southern hybridization. *Nco*I-digested genomic DNA was transferred onto membrane and probed with a radiolabeled 1.2-kb genomic fragment corresponding to the region immediately downstream of the *Trib3* stop codon. The expected size of the target fragment is 2.8 and 1.9 kb for the wild type and mutant alleles, respectively. (**C**) RT-PCR analysis of *Trib3* gene expression in P3 brain samples from wild type, heterozygous mutant and homozygous mutant littermate mice (n = 2 per genotype). *Gapdh* was amplified from the same samples as a positive control gene. The results from negative control reactions, which contained either total RNA that had not been subjected to reverse transcription (No RT) or water instead of template solution (No template), are also shown.
(TIF)

Figure S3 ***Trib1*** **(A) and** ***Trib2*** **(B) expression levels during mouse brain development.** RT-qPCR quantification of gene expression in wild type C57BL/6J mouse brain at embryonic day (E) 14, 15, 17 and 18, and at postnatal day (P) 0, 2 and 4. The mean expression level ± SEM at the indicated age is presented relative to the level of expression at E14 (n = 7 for E17, E18 and P0, n = 6 for E15, n = 5 for E14 and P2, and n = 3 for P4). Means marked with the same letter are not significantly different at the 5% significance level.
(TIF)

Table S1 **Composition of the diets used to study leucine deficiency.**
(PDF)
