# Supplementary figures and images for: Trib3 Is Developmentally and Nutritionally Regulated in the Brain but Is Dispensable for Spatial Memory, Fear Conditioning and Sensing of Amino Acid-Imbalanced Diet
Source: PLoS One. 2014 Apr 14;9(4):e94691. doi: 10.1371/journal.pone.0094691 (PMC3986210; doi:10.1371/journal.pone.0094691)

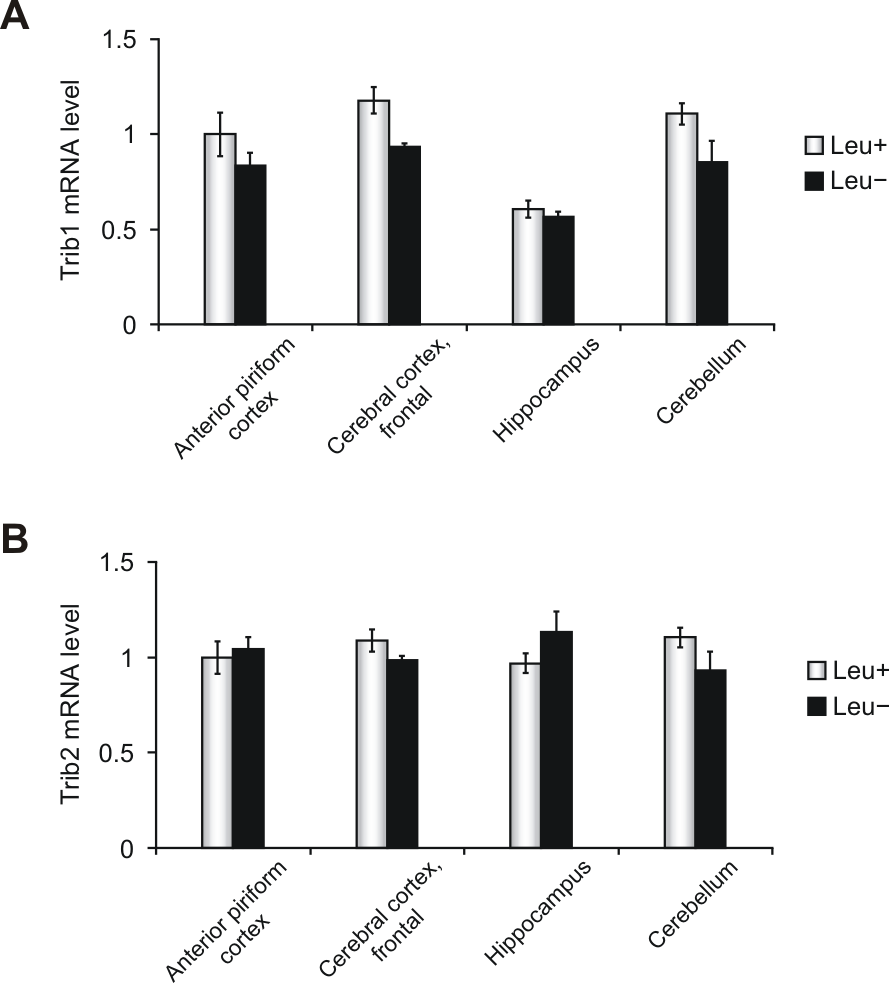

Supplement: Figure S1 — Trib1 (A) and Trib2 (B) expression levels in various regions of the adult mouse brain. Wild type mice consumed either a diet lacking leucine (Leu−; n = 5) or a corresponding control diet containing leucine (Leu+; n = 5), and, after 6 h of feeding, gene expression in the indicated brain regions was quantified by RT-qPCR. The results are presented as the mean ± SEM, and expressed relative to the level in the anterior piriform cortex of the control diet (Leu+) group. (TIF) [file pone.0094691.s001.tif]

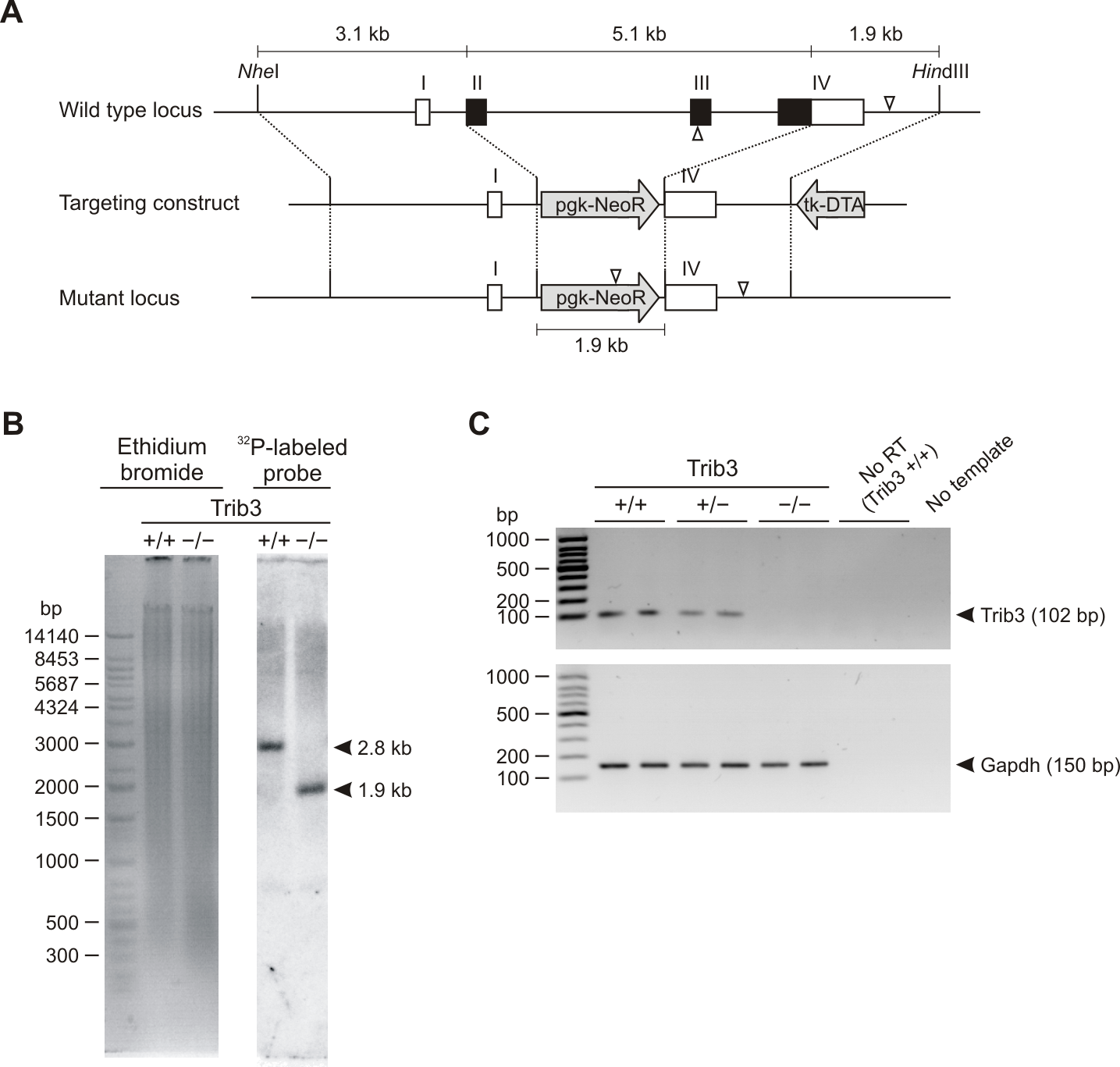

Supplement: Figure S2 — Targeted disruption of the mouse Trib3 gene. (A) Schematic representation of the gene targeting strategy used to generate the Trib3-deficient allele. Filled and unfilled boxes indicate exonal regions containing the Trib3 protein coding sequence and mRNA untranslated regions, respectively. The 5′ and 3′ homology arms (3.1 and 1.9 kb, respectively) for the homologous recombination event were selected to flank the genomic region corresponding to the Trib3 protein coding sequence. The homology regions were PCR-amplified and cloned into a targeting vector that contained a phosphoglycerate kinase promoter-driven neomycin resistance cassette (pgk-NeoR) for positive selection and a thymidine kinase promoter-driven diphtheria toxin A expression cassette (tk-DTA) for negative selection. The NcoI restriction sites that generate the genomic DNA fragments detected in panel B are indicated by unfilled vertical arrowheads. (B) Verification of the targeted disruption by Southern hybridization. NcoI-digested genomic DNA was transferred onto membrane and probed with a radiolabeled 1.2-kb genomic fragment corresponding to the region immediately downstream of the Trib3 stop codon. The expected size of the target fragment is 2.8 and 1.9 kb for the wild type and mutant alleles, respectively. (C) RT-PCR analysis of Trib3 gene expression in P3 brain samples from wild type, heterozygous mutant and homozygous mutant littermate mice (n = 2 per genotype). Gapdh was amplified from the same samples as a positive control gene. The results from negative control reactions, which contained either total RNA that had not been subjected to reverse transcription (No RT) or water instead of template solution (No template), are also shown. (TIF) [file pone.0094691.s002.tif]

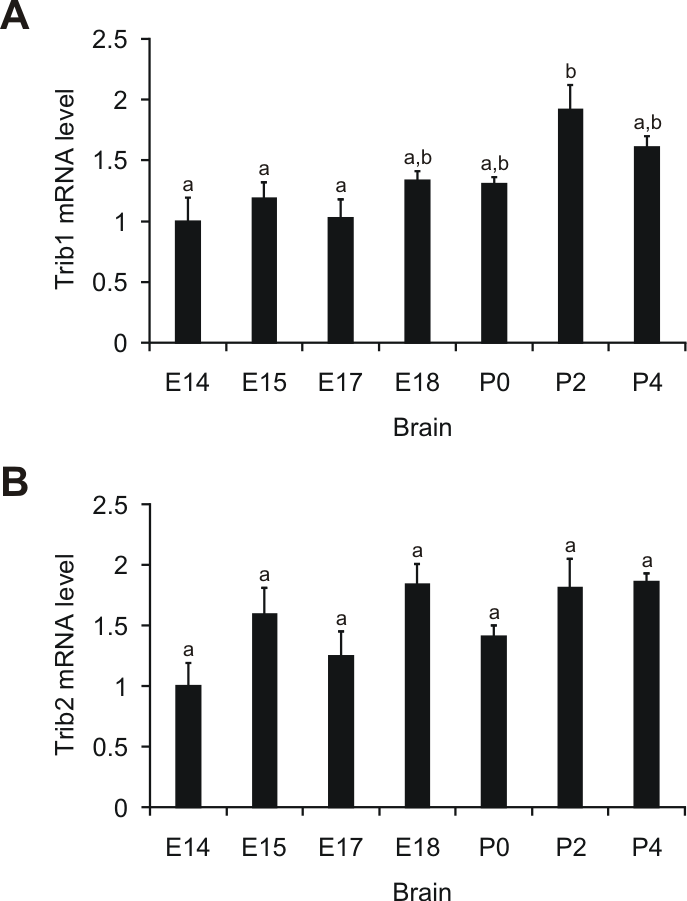

Supplement: Figure S3 — Trib1 (A) and Trib2 (B) expression levels during mouse brain development. RT-qPCR quantification of gene expression in wild type C57BL/6J mouse brain at embryonic day (E) 14, 15, 17 and 18, and at postnatal day (P) 0, 2 and 4. The mean expression level ± SEM at the indicated age is presented relative to the level of expression at E14 (n = 7 for E17, E18 and P0, n = 6 for E15, n = 5 for E14 and P2, and n = 3 for P4). Means marked with the same letter are not significantly different at the 5% significance level. (TIF) [file pone.0094691.s003.tif]
