# Supplementary material for: Trib3 Is Developmentally and Nutritionally Regulated in the Brain but Is Dispensable for Spatial Memory, Fear Conditioning and Sensing of Amino Acid-Imbalanced Diet
Source: PLoS One. 2014 Apr 14;9(4):e94691. doi: 10.1371/journal.pone.0094691 (PMC3986210; doi:10.1371/journal.pone.0094691)
Supplement: Table S1 — Composition of the diets used to study leucine deficiency. (PDF) [file pone.0094691.s004.pdf]

Table S1. Composition of the diets used to study leucine deficiency.

Ingredient (g)	Complete diet (control)	Leucine-deficient diet
L-Arginine	10	10
L-Histidine-HCl-H_2O	6	6
L-Isoleucine	8	8
L-Leucine	12	0
L-Lysine-HCl	14	14
L-Methionine	6	6
L-Phenylalanine	8	8
L-Threonine	8	8
L-Tryptophan	2	2
L-Valine	8	8
L-Alanine	10	10
L-Asparagine-H_2O	5	5
L-Aspartic acid	10	10
L-Cystine	4	4
L-Glutamic acid	30	30
L-Glutamine	5	5
Glycine	10	10
L-Proline	5	5
L-Serine	5	5
L-Tyrosine	4	4
Total amino acids	170	158
Corn starch	550.5	562.5
Maltodextrin 10	125	125
Cellulose	50	50
Corn oil	50	50
Mineral mix S10001	35	35
Sodium bicarbonate	7.5	7.5
Vitamin mix V10001	10	10
Choline bitartrate	2	2
Red dye, FD&C #40	0	0.025
Blue dye, FD&C #1	0.05	0
Yellow dye, FD&C #5	0	0.025
Total	1000.05	1000.05
